# Evaluation of Lithium Monitoring Practices for Patients in a Community Mental Health Team: An Audit Report.

**DOI:** 10.1192/bjo.2025.10642

**Published:** 2025-06-20

**Authors:** Abdulrahman Nafiu, Usman Siddique, Deepak Moyal

**Affiliations:** South West Yorkshire Partnership NHS Foundation Trust, Wakefield, United Kingdom

## Abstract

**Aims:** This audit aimed to assess the adherence of lithium monitoring practices within the Enhanced Teams of Folly Hall Community Mental Health Team (CMHT) to national and local guidelines. By identifying gaps and areas for improvement, the audit sought to enhance patient safety, optimize lithium therapy outcomes, and support service improvements.

**Methods:** A retrospective audit was conducted using data from 18 patients actively prescribed lithium. Information was collected from medical care plans, ICE (Integrated Clinical Environment) laboratory reports, and progress notes in SystmOne. The audit measured compliance with national (NICE NG181) and local monitoring standards, including:

Serum lithium levels (every 3 months).

Renal function tests (every 6 months).

Thyroid function tests (every 6 months).

Calcium levels (every 6 months).

Side effect monitoring (at every review or at least every 6 months).

Data collection was facilitated via a standardized Microsoft Form, and compliance was categorized as fully met (91–100%), partially met (81–90%), or not met (<81%).

**Results:** Lithium monitoring compliance was suboptimal: only 44.4% of patients had their lithium levels checked every 3 months.

Renal and thyroid function tests showed better adherence, with 94.4% and 88.9% compliance, respectively.

Calcium monitoring was inadequate, with only 61.1% compliance.

Side effect monitoring was well-documented (100% compliance), and prompt action was taken for all patients experiencing side effects (66% had dose reductions, and 33% had lithium discontinued due to severe adverse effects).

Action was not taken for one patient with out-of-range lithium levels, highlighting a significant safety concern.

**Conclusion:** The audit revealed significant deficiencies in lithium level and calcium monitoring, posing potential risks to patient safety. While renal and thyroid function monitoring showed high compliance, lithium level checks were insufficient, particularly for long-term users. The findings underscore the need for improved monitoring adherence to prevent toxicity and optimize treatment efficacy.

Recommendations:

1. Professional reminders in clinic rooms outlining lithium monitoring schedules.

2. Establishing a lithium monitoring registry for centralized tracking.

3. Regular discussion in business meetings to reinforce monitoring schedules.

4. Designation of a lithium monitoring champion to oversee compliance.

A re-audit is also being planned to evaluate the impact of these interventions.

